# Media attention and Vaccine Hesitancy: Examining the mediating effects of Fear of COVID-19 and the moderating role of Trust in leadership

**DOI:** 10.1371/journal.pone.0263610

**Published:** 2022-02-18

**Authors:** Lulin Zhou, Sabina Ampon-Wireko, Xinglong Xu, Prince Edwudzie Quansah, Ebenezer Larnyo

**Affiliations:** School of Management, Jiangsu University, Zhenjiang, PR China; University Magna Graecia of Catanzaro, ITALY

## Abstract

Vaccination has emerged as the most cost-effective public health strategy for maintaining population health, with various social and economic benefits. These vaccines, however, cannot be effective without widespread acceptance. The present study examines the effect of media attention on COVID-19 vaccine hesitancy by incorporating fear of COVID-19 as a mediator, whereas trust in leadership served as a moderator. An analytical cross-sectional study is performed among rural folks in the Wassa Amenfi Central of Ghana. Using a questionnaire survey, we were able to collect 3079 valid responses. The Smart PLS was used to estimate the relationship among the variables. The results revealed that media attention had a significant influence on vaccine hesitancy. Furthermore, the results showed that fear of COVID-19 played a significant mediating role in the relationship between media and vaccine hesitancy. However, trust in leadership had an insignificant moderating relationship on the fear of COVID-19 and vaccine hesitancy. The study suggests that the health management team can reduce vaccine hesitancy if they focus on lessening the negative impact of media and other antecedents like fear on trust in leadership.

## Introduction

The COVID -19 disease caused by the novel beta-coronavirus has resulted in severe consequences and unparalleled levels of misery and unemployment [1]. Among the interventions used to contain the virus, protective behaviors have been crucial [2], and vaccination could be one of them. Nonetheless, the vaccination would not be effective unless it gained widespread acceptance, highlighting the need to assess people’s hesitation to the COVID-19 vaccine. History shows that refusing to get vaccinated will have adverse health consequences [2,3]. The delay and refusal to be vaccinated is a global concern, and the World Health Organization has classified it as one of the ten most severe health risks for 2019 [4]. This is mainly due to the fact that herd immunity for COVID-19 requires approximately (55% to 82%) uptake [5]. Vaccine hesitancy is the ’postponement in acceptance or unwillingness to accept a vaccine given the existence of vaccination services’ [6]. Identifying predictive factors for COVID-19 vaccination hesitancy is crucial to derive interventions to enhance acceptance.

The spread of anti-vaccination propaganda via social media has given vaccine hesitancy and urgency in light of the pandemic and hopes for universal vaccine acceptance [7]. Ghana has roughly 16 million (15.7 percent) internet users. This reflected a 50% penetration rate, implying that almost half of the population had an internet connection in 2021 [8]. In the third quarter of 2020, 83.9 percent of the people connected to the internet in Ghana used WhatsApp. Facebook was also a popular social media platform, mentioned by approximately 71 percent of internet users.

Furthermore, YouTube represented 69.7 percent of the total internet users in the country [9]. However, over-reliance on media information has played a critical part in the emergence of fringe beliefs detrimental to public health. Burki [10] disclosed that paying much attention to the media Glanz, Wagner [11] found that web-based social media intervention increases early childhood immunization. Though the vaccine is regarded globally as a significant public health achievement, some media messages abound with skepticism, and falsehood about vaccination is likely to affect vaccine hesitancy. The current study, therefore, hypothesized that; *H1*: *Media attention has a significant influence on COVID-19* vaccine hesitancy. *H2*: *Media attention significantly influences fear of COVID-19 positively*.

Fear in research and vaccines (e.g., concerning the negative side effects and other adverse events) has become a commonly reported reason for vaccine hesitancy [12,13]. In a study piloted in Vietnam, [14] discovered that fear of contracting a disease positively impacts vaccine uptake among health workers. Savas and Tanriverdi [15] findings in Turkey showed the level of anxiety to be significant in participants who believed the vaccine was risky among healthcare workers. The studies stipulated above failed to examine the extent to which fear of COVID-19 could facilitate the relationship amid media attention and refusal of the COVID-19 vaccine. It is therefore hypothesized that; *H3*: *Fear of COVID-19 has a significant influence on vaccine hesitancy*, *H4*: *Fear of COVID-19 will play a mediating role in the relationship between media and vaccine hesitancy*

Trust is also attributed to the hesitancy of vaccines. Wang, Zhou [16] examined the impact of trust on the intention to vaccinate among community nurses and parents in Hangzhou province of China. Their findings established a negative relationship between mistrust and the intention to receive a vaccination. Mellis, Kelly [17] study displayed that the level of trust could affect the distribution of the Covid-19 vaccine. Besides providing additional insights into vaccine effectiveness, trust in leadership is an essential factor [18]. The current study believes that when trust in leadership upsurges from low to high or moderate, individuals will be more likely to avoid the delay and refusal to be vaccinated, as their fear of COVI-19 will reduce.

Conversely, individuals with a low level of trust in their leaders would be more likely to refuse to be vaccinated due to the increased fear. It is, however, posited that; *H5*: *Trust in leadership will moderate the relationship fear of COVID-19 and vaccine hesitancy*.

**Fig 1:**
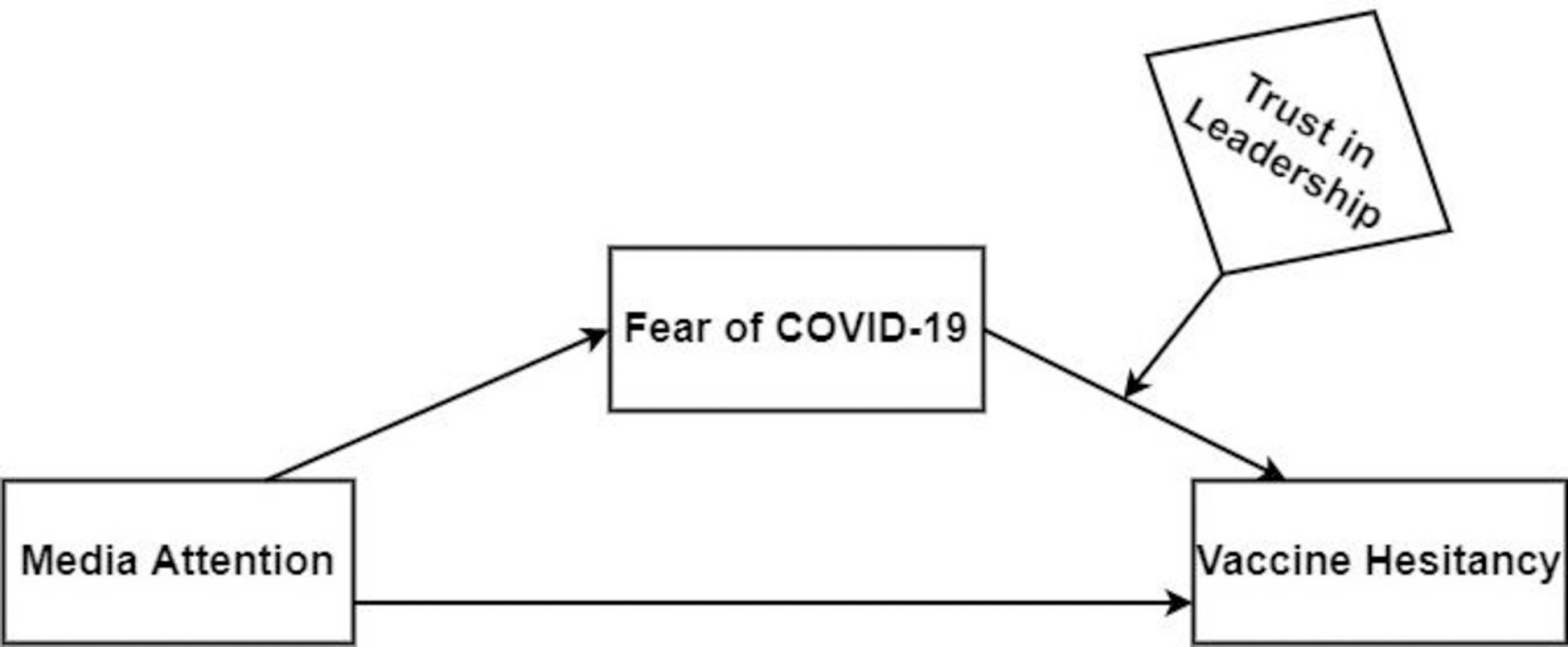
Conceptual model depicting the mediated and moderated effects of fear of COVID-19 and trust in leadership in the association between media attention and vaccine hesitancy.

Understanding the media’s role in receiving COVID-19 vaccination is critically important with the forthcoming need for worldwide COVID-19 vaccination programs. Addressing the determinants of COVID-19 vaccine hesitancy by employing media attention [19,20], fear [21,22], and trust [23,24] are key at the policy level to prevent the unwillingness or refusal to vaccinate, notwithstanding vaccine availability. Nevertheless, no literature has examined fear of COVID-19 as the mechanism through which media attention affects vaccine hesitancy in one model. The present study has covered some novel aspects of media and its effects on COVID-19 vaccine hesitancy from both practical and academic perspectives. The present study further contributes to literature to examine the mediating effect of fear of COVID-19 in the relationship between media attention and vaccine hesitancy. The current research again extends existing studies by assessing the interactive impact of trust in leadership in the relationship between fear of COVID-19 and COVID-19 vaccine hesitancy, which is, has been ignored in past literature. Findings from this study may provide insight to design health promotion programs that may help improve confidence in vaccination among the rural people of Ghana. Fig 1 shows a diagrammatical presentation of the aforementioned variables effects on COVID-19 vaccine hesitancy in Ghana.

## Materials and methods

### Study area

Approximately 45% of Ghanaians live in a rural community [25]. According to the most recent data (2010), the Amenfi central district had 69,014 people, with 52 percent of the population male and 48 percent female. The data also reveals that the vast majority of the population (72.5 percent) resides in rural areas. The district is ethnically unified, with Akans constituting the vast majority (82 percent). Seventy-seven percent (77.4%) of the employed population is recruited as skilled agricultural, forestry, and fishery workers. Six-point seven percent (6.7%) are employed in service and sales work, 5.1 percent is are engaged in craft and general market activities, and 3.5 percent are employed as managers, professionals, and technicians, according to the latest available data.

### Data collection method

The study implemented a population-based cross-sectional survey design to evaluate the influence of media attention on COVID-19 vaccine hesitancy from (8) communities (Achichire, Anakum, Sureso, Amuni, Wassa Ankwaso, Juabo, Aserewadi, and Ankasie) within the Wassa Amenfi Central district of Ghana. In short, 3079 residents aged 18–65 years in eight rural communities in the Western region were investigated using a multi-stage stratified cluster sampling method. Individuals who were not eligible for the COVID-19 vaccine were excluded from the study. This includes older age (persons older than 65 years), those who have had organ transplants, stem cell transplants, and cancer. Pregnant women, lactating mothers, those with primary immunodeficiency, people treated with immunosuppressive medications were excluded. This inclusion and exclusion strategy was in conformance to the eligibility criteria of COVID-19 vaccination provided by Dooling [26]. The study’s eligibility requirements were also included on the consent form. The eligibility included prospective participants answering no to the following two questions: 1. Are you above 65 years of age? Yes, or No?; 2. Are you pregnant Yes, or No?; 3. Are you currently breastfeeding? Yes, or No?; 4. Do you have any of these conditions at the moment? Cancer, Tuberculosis, HIV, or any other immunocompromised disease …. Yes, or No?; (2) Will you be able to take part in four data collection to be performed? Yes or No? Have you had an organ transplant recently? Yes, or No? To win the trust of the respondents and to avoid the feeling of stigmatization, they were assured that their personal information would be kept private and that the information would only be used for academic purposes. The data collection exercise took place from April 2020 to December 2020. This was followed by the training of ten field assistants, data collection instrument piloting, and questionnaire revision based on-field challenges. In each neighborhood, one house was chosen at random from the first to the third on the list. Only individuals above age 18 were selected as respondents; however, if the individual above age 18 of a selected household was unavailable, the researchers moved to the next house. A nominated home was only substituted if no adults were found after a third visit. When there were multiple households in a dwelling, none were chosen at random, for it was assumed that all of the families shared similar features. In addition, if no one was found in a specific house or no member of that house was ready to be involved, the next house was chosen. At the end of the data collection exercise, we obtained 3079 usable completed questionnaires (valid response rate of 87.5%.), comprising 410, 318, 397, 401, 421, 389,312, and 431 for Achichire, Anakum, Sureso, Amuni, Wassa Ankwaso, Juabo, Aserewadi, and Ankasie communities respectively.

### Consent to participate

The research was carried out per the national ethical guidelines and regulations. Participants were notified of the study’s purpose and that any data they provided would be kept strictly confidential. A consent form includes ‘My participation is entirely voluntary, and I may withdraw at any time without penalty or loss of benefits to which I am entitled. Similarly, the participants were assured that any information they provided will be kept confidential and will not be released without my consent, except as required by law. Approval for the study was received from the Ghana Health Service ethical commute with the number GHS-ERC: 32/16/20.

### Measures of instruments and hypothesis

The questionnaire included five constructs; demographic information, COVID-19 vaccine hesitancy, trust in leadership, fear of COVID-19, and media attention. Demographic information contains age, gender, occupation, and marital status. The scales were measured with a 7-point Likert scale (1 = strongly disagree, 7 = strongly agree).

#### Vaccine hesitancy

Vaccine hesitancy is a dynamic research field with various terms, concepts, and measurements [27]. The fifteen elements of the vaccine hesitancy questionnaire were re-worded correctly to make it suitable for all survey respondents [28]. For instance, “I would recognize myself as willing to receive COVID-19 vaccine” was rephrased, as “I would identify myself as willing to get a COVID-19 vaccine.” Each item was graded on a 1 (strongly disagree) to 7(strongly agree) scale. Higher scores specify greater hesitancy. Three items (VH2, VH8, and VH9) were deleted because they loaded onto different components during the factors loadings assessment.

#### Trust in leadership

In this study, trust in leadership is defined as a leader’s capacity, skill, and reliability in carrying out activities. We adapted items from Liu, Cheng [29] to measure trust in leadership. Six items were modified from the literature as mentioned above to measure how respondents’ trust affects the hesitancy of the COVID-19 vaccine. Examples of such items include “trust in the government of Ghana health experts” and “trust in the safety of COVID19 vaccine”. With a Cronbach alpha of 0.8. The researchers deleted two of the items since they loaded poorly during the confirmatory factor analysis.

#### The Fear of COVID-19

Ahorsu, Lin [30] proposed the scale, which was then adapted to the Ghanaian context for this analysis. It is a seven-item one-dimensional scale. A 5-point Likert-type scoring system is used (ranging from 1: Strongly disagree to 5). The original scale’s item factor weights range from.66 to.74, and item-total correlations range from.47 to.56. The scale’s Cronbach’s alpha internal consistency coefficient is 0.82. “I become nervous when I think about COVID-19,” for example, is an example of fear of COVID-19. “When I think about COVID-19, my hands get clammy.”

#### Media attention

People’s ability to actively commit cognitive effort to specific media messages is media focus [31]. Five items were adopted to measure media attention’s effects on COVID-19 hesitancy among nurses and midwives in Ghana. Examples of such items include “Seeking information from the television, internet, and newspapers help me to gives me ideas about how to discuss the issue of COVID-19 with others.”

#### Control variables

Variables such as gender, age, religion, and occupation are suggested to affect the acceptability of vaccines among respondents [32]. We, therefore, decided to control them in our model.

## Data analysis

The data was evaluated with the Statistical Package for Social Science (SPSS) 12.0 and the Smart PLS 3.0. To measure the fitness of the hypothesized model, the structural equation modeling (SEM) analysis was performed. The research was focused on stages [33] that included (a) measurement model assessments and (b) examination of the hypothesized models. Model fit was evaluated using chi-square statistics. The relationship’s significance was determined using resampling with substitution from the original sample using a nonparametric bootstrapping method to estimate regression coefficients [34]. The bootstrapping method generates probability values that demonstrate the path coefficients’ consistency [35]. The smart PLS can connect different latent variables by estimating a network of interaction effects based on a theoretical model [36]. A two-stage estimation technique was used in the Smart PLS. The first stage uses a sequence of interactive measures to estimate the dependent variable’s score and outer loadings and outer weights for evaluating constructs [37]. The second stage involves calculating path coefficients between the latent variables.

### Common method bias test

We employed various steps to handle common method variance in our data. While designing and distributing the questionnaires, we followed the proposed steps of Podsakoff, MacKenzie (38). The steps included randomizing the items’ order and issuing reports to the respondents that the research was solely for academic purposes. Also, we informed the respondents that they should feel free to choose any answer they deemed fit and that there was no right or wrong answer. Furthermore, Podsakoff, MacKenzie [38]; Podsakoff, MacKenzie [39] highlight that participants are more motivated to be more accurate if they believe the information provided will benefit them or the organization, and encouraging feedback may also motivate greater accuracy. For this reason, we assured the respondents that the information they provided would enable the design of specific policy guidelines to encourage management support, increase specific motivation and achieve high job satisfaction. Again, we kept the survey items short to minimize redundant measures and overlap that helped the participants to give more accurate responses. Respondents were assured that their responses would remain anonymous. According to Johnson and Fendrich [40], is process assists respondents in projecting an objective image of themselves. We further employed Harman’s one-factor test to detect threats of common method bias. An unrotated, principal component factor examination of all measurement items showed eight factors with eigenvalues above one. The first factor demonstrated 38.36% of the total variance, less than 50%, with all variables accounting for 73.56 percent of the total variance. These results indicate that common method bias was not a problem.

## Results and discussion

### Demographic characteristics of the participants

Approximately 4,200 questionnaires were distributed to participants, out of which the first respondents received 3518. Questionnaires were completed anonymously by respondents, but the workers indicated their age, gender, occupation, and religion. Out of the 3518 questionnaires, 3079 were completed without errors by the participants’ showing a valid response rate of 87.5%. With this, 1302 (42.2%) were females, and 1777 (57.7%) were males. The majority of the respondents between the ages 18 and 60 was 2919 (94.8%), whereas only a few participants, 160 (5.1%), were above 60 years, indicating the district has a lot youthful population. In the case of occupation, 701(22.7%) were engaged in small scale mining, 708 (22.9%) were farmers, and 483 (15.6%) were government workers, 301 (9.7%) were into commercial driving, whereas 686 (22.3%) were not employed. Out of the total respondents, 2298 (74.6%) were Christian, 318 (15.2%) belong to the Islamic region, whereas either traditional believers, 103(3.3%), Buddhism 59 (1.9%), and non-religious 301 (9.7%).

### Measurement model

The data were assessed for internal consistency reliability, convergent, and discriminant validity [41]. The factors loadings, Cronbach alpha, average variance extracted (AVE), and composite reliability [41,42] that satisfy the acceptance criteria for inclusion were used to examine the measurement model are shown in Table 1. The Cronbach’s alpha is used to assess internal reliability; the findings of the Cronbach alpha ranged from 0.953 to 0.910, which is greater than 0.7 according to the recommended threshold indicates sufficient reliability. The composite reliabilities are greater than 0.50; this shows that the convergent validity is sufficient [43]. All of the measures were above 0.70 and statistically relevant at the 0.01 stage.

**Table 1:** Factor loadings, reliability and validity analysis.

Variables	Factor loadings	Cronbach’s Alpha	Composite Reliability	Average Variance Extracted (AVE)
FoC1	0.990	0.953	0.953	0.721
FoC2	0.902			
FoC3	0.727			
FoC4	0.704			
FoC5	0.892			
FoC6	0.856			
FoC7	0.971			
FoC8	0.69			
MA1	0.719	0.910	0.913	0.683
MA2	0.819			
MA3	1.041			
MA4	0.693			
MA5	0.814			
TL1	0.782	0.912	0.915	0.731
TL2	0.744			
TL4	0.879			
TL6	0.993			
VH1	0.706	0.948	0.949	0.728
VH2	1.035			
VH3	0.799			
VH4	0.872			
VH5	0.838			
VH6	0.902			
VH7	0.778			

Abbreviation: FoC, fear of COVID-19; MA, Media attention; TL, trust in leadership; VH, COVID-19 vaccine hesitancy.

Table 2 shows the discriminant validity of the components fear of COVID-19, media attention, leadership trust, and vaccination hesitation. The discriminant validity was calculated using the square root of the AVE and cross-loading matrix of 0.849, 0.827, 0.855, and 0.853, respectively. Hair [44] disclosed that for adequate discriminant validity, the square root of a construct’s AVE should be bigger than its associated constructs. Interestingly, the diagonal values are greater than those in related columns and rows, which satisfies the discriminant validity [45].

**Table 2:** Fornell-Larcker criterion.

Variables	Fear of COVID-19	Media Attention	Trust in Leadership	Vaccine Hesitancy	
Fear of COVID-19	***0*.*849***				
Media Attention	0.453	***0*.*827***			
Trust in Leadership	-0.528	-0.546	***0*.*855***		
Vaccine Hesitancy	0.439	0.443	-0.538	***0*.*853***	

The bolded values in the diagonals of the respective variables denote the square correlations between the variables.

### Hypothesis testing

#### Testing of the effect of media attention on vaccine hesitancy

The study used the structural equation model in SMART-PLS version 3.2 software to test the hypothesized associations illustrated in the conceptual framework (Fig 1). The study followed the procedures recommended in related literature for testing a structural model containing the mediation and moderation variables [46–49]. Finally, we assessed the structural model for model fitness using Chi-square, standardized root mean square residual (SRMR), squared Euclidean distance (d_ULS), geodesic distance (d_G), and Normed fit index (NFI). The results (SRMR = 0.042, d_ULS = 0.539, d_G = 0.267, Chi-Square = 827.001, and NFI = 0.932) showed that data had an acceptable model fit [50]. First, we examined the main effect model (Fig 1), which involves media attention on vaccine hesitancy. The results of the main effect model showed that media attention has a significant positive effect on vaccine hesitancy among rural inhabitants (β = 0.392, p < 0.001). The results indicate individuals who were increasingly receiving false messages through media platforms are more likely to be hesitant in getting the COVID-19 vaccine and hence, offer support for H1.

### Testing of the mediating effect of fear of COVID-19 in the relationship between media attention and vaccine hesitancy

**Fig 2:**
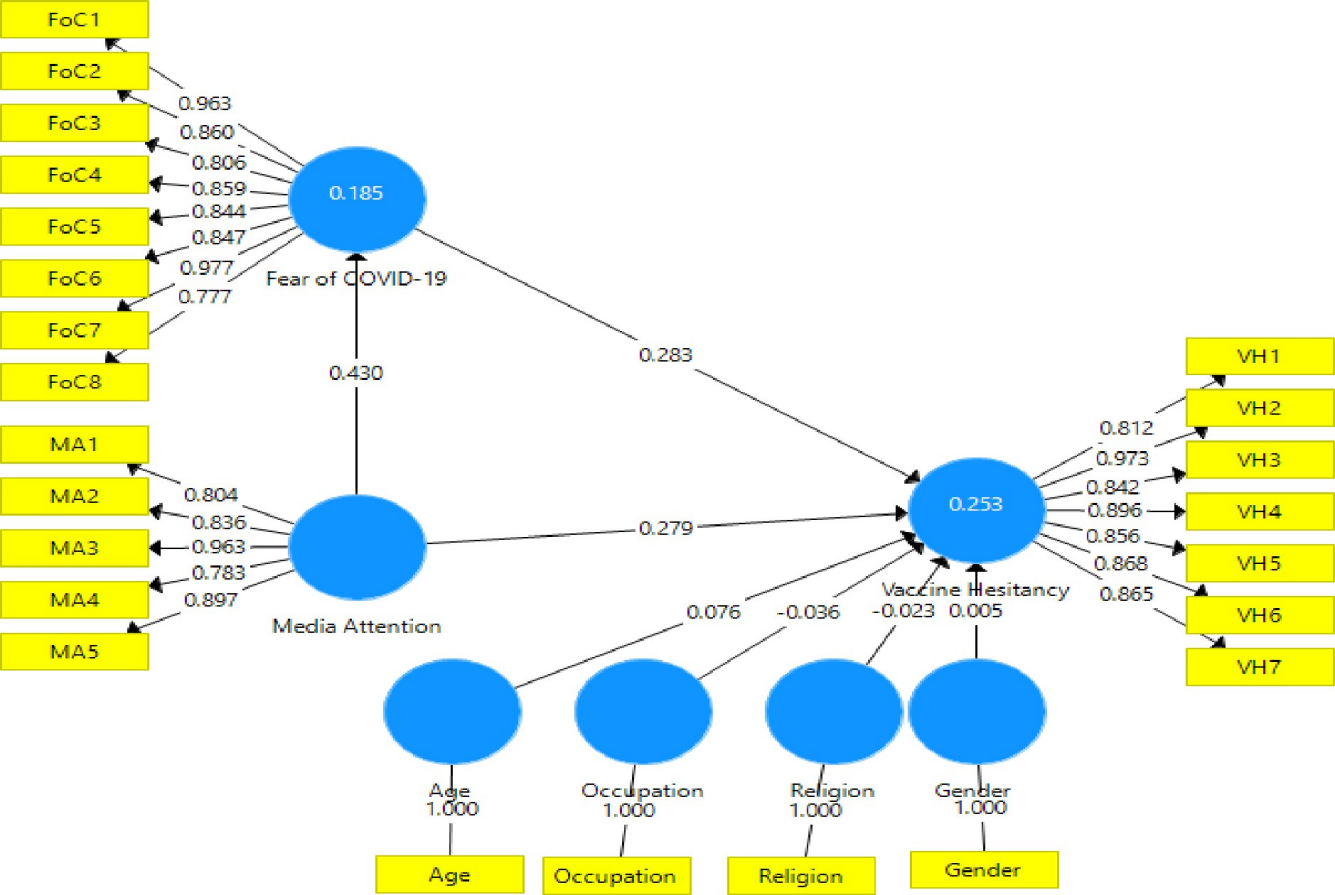
Structural mediation model showing Fear of COVID-19 as a mediator in the relationship between media attention and vaccine hesitancy.

**Fig 3:**
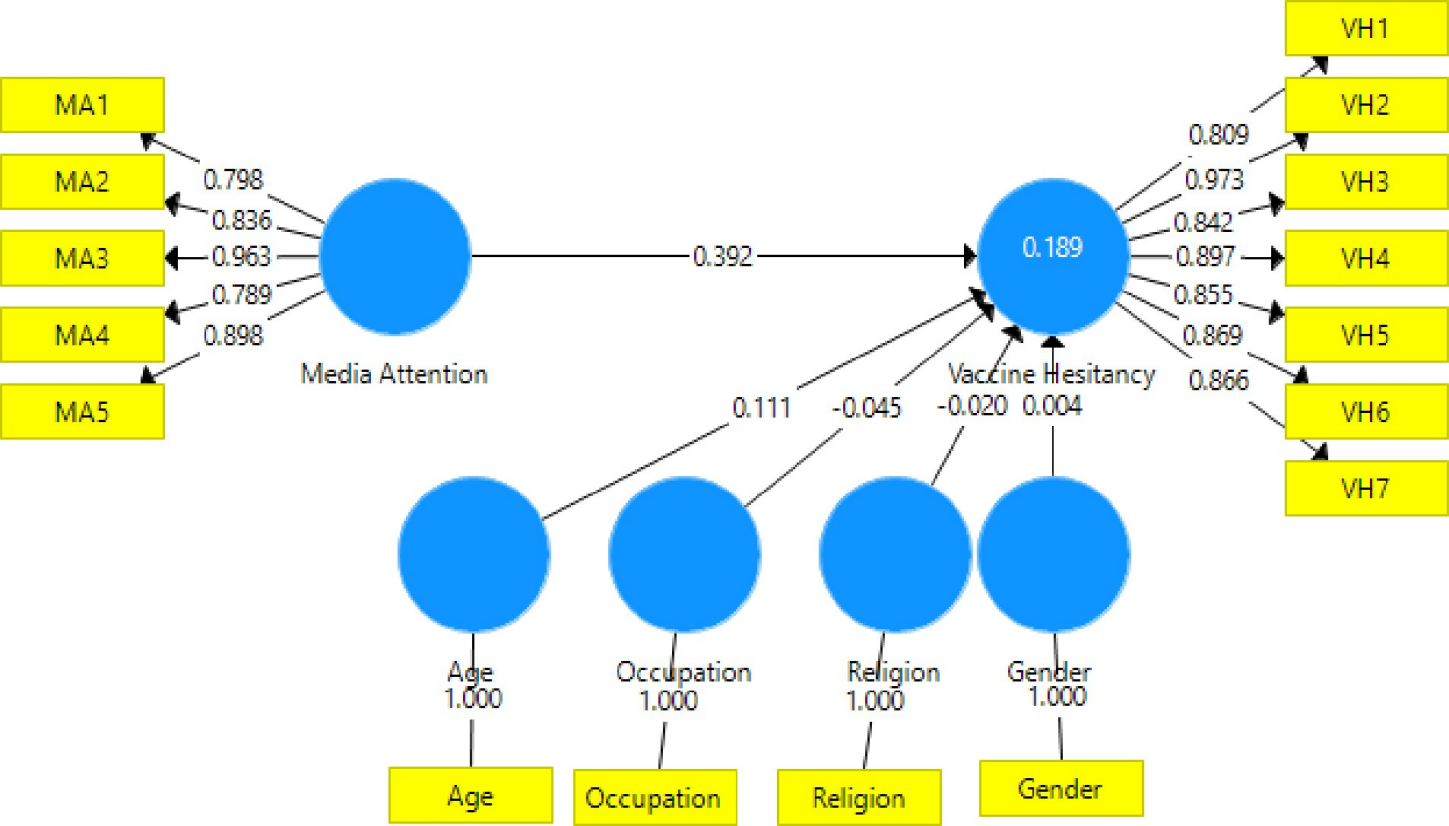
Main effect model showing the direct effect of media attention on vaccine hesitancy.

Next, the study performed the structural mediation model (see Fig 2), where fear of COVID-19 (FoC) was added to the main effect model (Fig 3), and the results are presented in Table 3. The study utilized a bootstrapping method with a 5000-sample bias-corrected 95% confidence intervals (CI). Relying on the rule of thumb for bootstrapping, an insignificant effect is obtained where zero falls within the 95% CI. In contrast, a significant association is achieved where zero does not fall within the 95% CI. Specifically, while controlling for age, gender, religion, and occupation, media attention still had a significant positive effect (β = 0.43, p < 0.001) on COVID-19 vaccine hesitancy even when fear COVID-19 was introduced to the model. The findings, therefore, provide additional support for H1. Media attention influences vaccine hesitance, thus supporting H2. Fear of COVID-19 had a significant positive influence on vaccine hesitancy, which did support H3. The effects of fear of COVID-19 in the relationship between media attention and COVID-19 hesitancy showed partial mediating effects (β = 0.283, p < 0.001), supporting hypothesis H4. That is, when people pay more attention to media information, they are more likely to be fearful of COVID-19, which could discourage them from being vaccinated.

**Table 3:** Results from the structural mediation model.

Path	Original Sample (O)	Sample Mean (M)	Standard Deviation (STDEV)	T Statistics (|O/STDEV|)	2.5%	97.5%	P Values
Age -> VH	0.076	0.076	0.037	2.035	0.001	0.146	0.042
FoC -> VH	0.283	0.282	0.049	5.745	0.19	0.38	0.000
Gender -> VH	0.005	0.007	0.035	0.133	-0.061	0.075	0.895
Occupation -> VH	-0.036	-0.036	0.033	1.105	-0.099	0.029	0.269
Religion -> VH	-0.023	-0.02	0.034	0.657	-0.086	0.049	0.511
MA -> VH	0.279	0.286	0.05	5.542	0.185	0.385	0.000
MA -> FoC	0.43	0.431	0.045	9.549	0.346	0.521	0.000
MA -> FoC -> VH	0.122	0.121	0.025	4.971	0.078	0.174	0.000

Abbreviation: FoC, fear of COVID-19; MA, Media attention; TL, Trust in leadership; VH, COVID-19 vaccine hesitancy.

### Testing of the moderating effect of trust in leadership on the relationship between fear of COVID-19 and vaccine hesitancy

**Fig 4:**
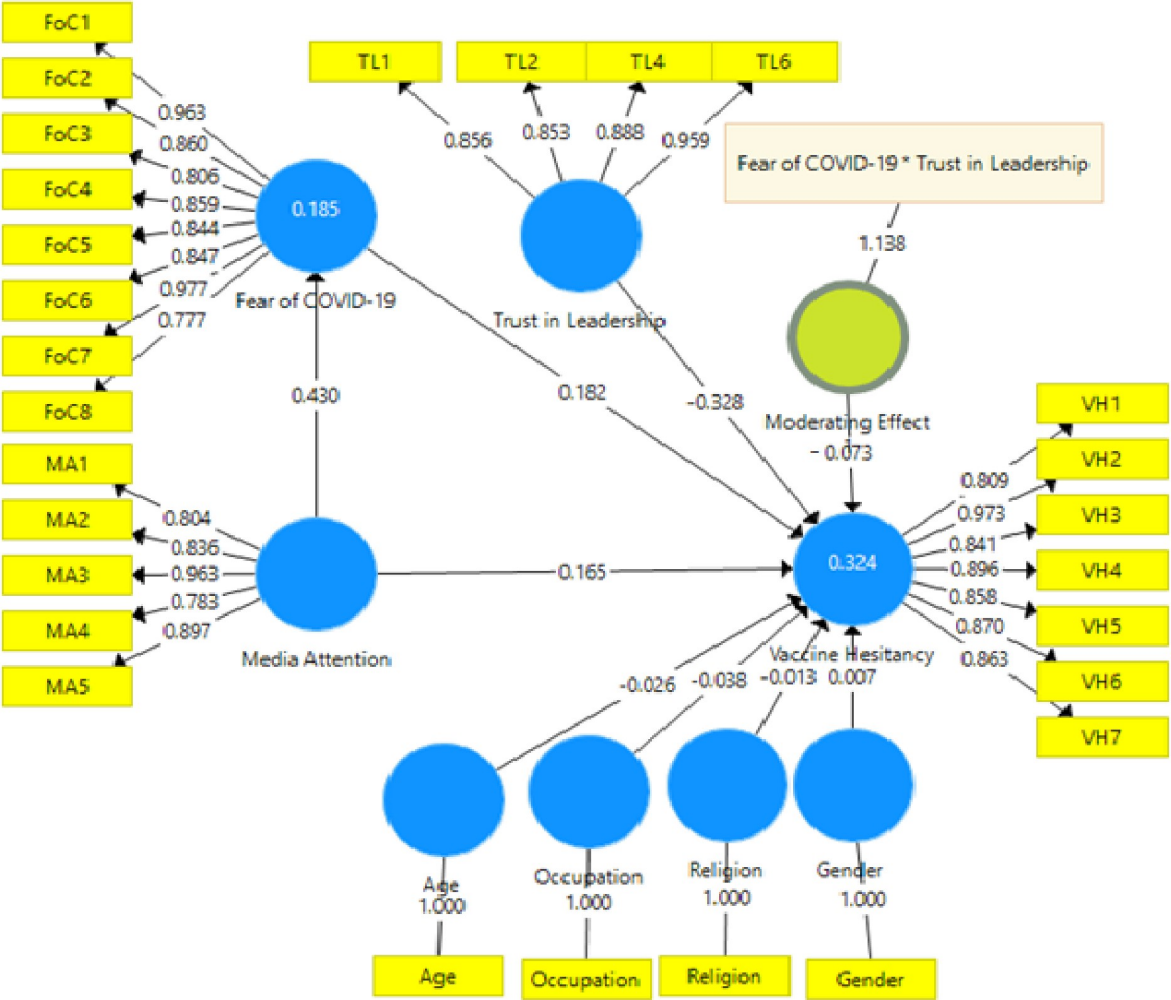
The structural moderating effects of trust in the relationship between fear of COVID-19 and vaccine hesitancy.

The study moved further to examine the moderating role of trust in leadership on the relationship between fear of COVID-19 and COVID-19 vaccine hesitancy after the structural mediating analysis. The model as shown in Table 4 established that moderating effects of trust in leadership on the relationship between fear of COVID-19 and COVID-19 vaccine hesitancy is negative and significant (-0.090, p < 0.001). That is, the positive relationship between fear of COVID-19 and vaccine hesitancy is weakened by people’s trust in leadership, as graphically presented in Fig 4. This finding did not support H6. This indicates that when trust in leadership is high, the positive effect of fear of COVID-19 on vaccine hesitancy is reduced. However, the impact of fear of COVID-19 becomes stronger when people have less trust in their leaders. Interestingly, the findings presented in appendix Table 1 indicate media attention had a significant positive relationship with vaccine hesitancy, hence supporting H1. In addition, media attention again had a significant positive effect on fear of COVID-19, which also endorses H2.

**Table 4:** Results of the moderating effect from figure.

Path	Original Sample (O)	Sample Mean (M)	Standard Deviation (STDEV)	T Statistics (|O/STDEV|)	2.50%	97.50%	P Values
*Controls*							
Gender -> VH	-0.006	-0.008	0.036	0.175	-0.078	0.064	0.861
Age -> VH	-0.01	-0.01	0.035	0.279	-0.08	0.058	0.78
Education -> VH	-0.042	-0.042	0.031	1.331	-0.102	0.02	0.183
Occupation -> VH	0.064	0.064	0.035	1.853	-0.003	0.133	0.064
Religion -> VH	-0.026	-0.027	0.033	0.787	-0.092	0.039	0.431
*Direct paths*							
FoC -> VH	0.207	0.208	0.049	4.217	0.111	0.302	0.000
TL -> VH	-0.275	-0.276	0.048	5.706	0.181	0.372	0.000
Interaction							
FoC *TL-> VH	-0.09	-0.09	0.023	3.886	0.048	0.138	0.000

Abbreviation: FoC, fear of COVID-19; MA, Media attention; TL, trust in leadership; VH, COVID-19 vaccine hesitancy, and MA*FoC is the interaction between media attention and fear of COVID-19.

## Discussion

Vaccination has become one of the most remarkable progress in public health. As a result, researchers have been working persistently to create and test novel vaccines to protect humans from COVID-19 [51]. Researchers’ efforts will be futile if the general public refuses to be vaccinated. The study examines the role of media attention and vaccine hesitancy. Further, the mediating effect of fear of COVID-19 on the relationship amid media attention and vaccine hesitancy represents another contribution of this study. The moderating impacts of trust in leadership on the association between fear of COVID-19 and vaccine hesitancy are studied.

The relationship between media attention and COVID-19 vaccine hesitancy was significantly positive. In other words, exposure to positive news about COVID-19 in the media positively influences the decision to accept the vaccine, while exposure to misleading or negative information about the virus can lead to skepticism towards the vaccine. According to the findings, paying so much attention to details on Covid-19 from the media could significantly impact public opinion as to whether or not they would want to be vaccinated. Buller, Walkosz [52] described how media users appeared to be preferentially influenced by narrative stories favoring vaccine reluctance. Brief exposure to websites and social media posts criticizing vaccinations has been shown to influence risk perception and vaccine reluctance and discourage vaccination [20]. Viewing or hearing such content can have a detrimental effect on users’ vaccination intentions [53]. Nyhan, Reifler [54] showed that vaccine-hesitant parents subjected to vaccination myths on social media were more persistent in their anti-vaccination beliefs and had fewer plans to vaccinate their children.

The study confirmed a significant positive relationship between media attention and fear of COVID-19. Interestingly, fear of COVID-19 also had a significant influence on vaccine hesitancy. The implication of the findings is that continuous misinformation from the media platforms could trigger the fear of COVID-19 and the feeling of unnecessary public panic, which could lead to vaccination delay and refusal.

Another section of the study investigated the mediating effect of fear of COVID-19 in the relationship between media and vaccine hesitancy. The analysis also reveals a partial mediating impact of fear of COVID-19 in the relationship between media attention and vaccine hesitancy. Policymakers must immediately begin considering methods to combat the tendencies identified in this study. The current study’s findings are supported by the findings of Nyan et al. [55] in Greece, who established negative news from the media to be associated with fear.

According to this study’s results, the moderating role of trust in leadership revealed an insignificant positive in the relationship between fear of COVID-19 and vaccine hesitancy, which is inconsistent with our expectations. Although the interaction between fear of COVID-19 and trust in leadership has a negligible effect on vaccine hesitancy, the results raise some interesting concerns. This study also suggests that in light of non-statistically significant interactions among predictive variables, it will not be erroneous to interpret the main effects. The outcome of the main impacts of trust in leadership on vaccine hesitancy was significantly negative. This is possible because people look to leaders to be calm and deliberate in their decisions and actions regarding their acceptability of the vaccine. In other words, individuals with increased trust in leaders are less likely to refuse and delay receiving COVID-19 vaccination. The finding of our study is consistent with previous studies of studies [55,56].

Even though our findings can only show the relationship between media attention, fear of COVID-19, Trust in leadership, and vaccine hesitancy in rural Ghana, it suggests implications relevant to health public experts and policymakers. Scholars interested in studying factors that influence vaccine acceptability will learn from this study by replicating it differently in urban areas. Principally, the research has established that media attention significantly affects vaccine hesitancy. Furthermore, the study contributes to the body of knowledge by demonstrating that fear of COVID-19 played a significant mediating role in explaining why media attention influences vaccine hesitancy. With theoretical direction, this study’s findings determine how to increase vaccine acceptability by focusing on the impact of media and religion, and subjective norms on vaccine hesitancy.

The current research revealed that exposure to misleading information on COVID-19 vaccination through media platforms could increase COVID-19 vaccine hesitancy among the rural population. Health policymakers are encouraged to use print, social, and electronic media to raise awareness about the benefit of vaccinating against the COVID-19 virus. The use of graphics, video material, narrative statements, and other forms of media can be beneficial, as they can help stimulate positive beliefs. The e-guidelines are also needed to combat fake news, particularly prevalent on social media. Health professionals should collaborate with the mass media management team to provide teletherapy as a subject matter to their viewers. Second, the mediating effects of fear of COVID-19 significantly mediated the relationship between media attention and vaccine hesitancy. To disrupt the vicious circle of anxiety and panic surrounding COVID-19, persistent and supportive actions are required. Diverse strategies through community engagement and assistance could be part of methods necessary at individual and collective levels. On another note, an increase in trust in leadership negatively affects vaccine hesitancy in Ghana. All stakeholders, including health care providers, community leaders, policymakers, and the media, must present vaccine-related issues transparently to assure the general public’s safety. Other interventions aimed at improving positive vaccination attitudes and actions across the public may also be required.

## Conclusion

COVID-19 disease is a serious health problem affecting millions of lives globally. The implication of COVID-19 vaccination is only feasible if the individual avoids the delay and refusal and responds positively towards the acceptability of the vaccine recommended by the government. However, literature remains limited in Ghana regarding the factors influencing the hesitancy of COVID-19 vaccination in rural Ghana. This research examined the impact of influencing factors affecting the hesitation of COVID-19 vaccination among the rural population in Ghana. The study employed a survey technique and a well-structured questionnaire as the instrument for the data collection. The Statistical Package of Social Science (SPSS) and the Smart PLS software was used to analyze the data’s preliminary examination. The study revealed that media positively influenced the delay to accept COVID-19 vaccination among rural folks in Ghana. Fear of COVID-19 partially mediated the relationship between media and vaccine hesitancy. In the case of trust in leadership as a moderator, an insignificant relationship is shown. Since the data collection process is cross-sectional, two proposed methods for controlling common method bias have been carefully implemented: a. research procedure design and b. statistical tests. Nonetheless, the data may be subject to widespread process bias. As a result, the measurement prediction criterion variables should be segregated by time to establish a strong relationship between media and COVID19-vaccination hesitancy in future studies.

**Appendix:** Questionnaire for the study.

**Constructs**	**Description**
Vaccine hesitancy	
VH1	I will take a COVID-19 vaccine if offered
VH2	If the COVID-19 vaccine is available, I will want to get it as soon as possible
VH3	I would describe my attitude towards receiving a COVID-19 vaccine as Very keen
VH4	I would take the COVID-19 vaccine when it is available at my local pharmacy
VH5	I will take getting a COVID-19 vaccination; family or friends were thinking
VH6	I would describe myself as eager to get a covid-19 vaccine.
VH7	I will take COVID-19 vaccination because it is important
The Fear of COVID-19	
	I am most afraid of COVID-19.
FoC1	It makes me uncomfortable to think about COVID-19.
FoC2	My hands become clammy when I think about COVID-19.
FoC3	I am afraid of losing my life because of COVID-19.
FoC4	When watching news and stories about COVID-19.on social media, I become nervous or anxious.
FoC5	I cannot sleep because I’m worried about getting COVID-19.
FoC7	My heart races or palpitates when I think about getting COVID-19.
FoC6	I am most afraid of COVID-19.
Media Attention	
MA1	How often do you come to COVID 19 pandemic coverage from three types of media, including television, newspapers, and the Internet
MA2	How much attention does the respondent pay to news stories about COVID-19.
MA3	Seeking information from the television, internet, and newspapers helps me find out about COVID-19
MA4	Seeking information from the television, internet, and newspapers helps me to observe how others deal with climate change,” “gives me ideas about how to discuss the issue of COVID-19 with others,”
MA5	Seeking information from the television, internet, and newspapers “helps me figure out how I can be prevented from getting COVID-19.
Trust in leadership	
TL1	I know what exactly my leaders will do in times of difficult situation
TL2	My leaders have my best interest in mind
TL3	My leaders behave in a consistent manner
TL4	My leaders are likely to protect me
TL5	My leaders are likely to protect me
TL6	My leaders know exactly what they are doing
